# Design of a special rigid wheel for traversing loose soil

**DOI:** 10.1038/s41598-022-27312-6

**Published:** 2023-01-04

**Authors:** Mogeeb A. Elsheikh

**Affiliations:** Department of Production and Mechanical Design Engineering, Faculty of Engineering, Port Said University, Port Said, Egypt

**Keywords:** Engineering, Mechanical engineering

## Abstract

Wheels play an important role in mobile robotics, wheelchairs and vehicles and represent an ideal solution for traversing rigid ground due to higher efficiency. Through traversing loose soil, the rigid wheels lose traction because of sinking and higher slip ratios. Therefore, the study suggests a new rigid wheel with a distinguished perimeter to increase mobility demands to overcome the previous inevitable concerns and clarifies its full detailed design. The lateral undulation locomotion of snakes inspired the author to introduce a new simple and affordable wheel design. The optimum values of the limbless creature movement on the sand are reflected in the geometrical parameters of the wheel, amplitude to wavelength ratio. In addition, the experimental work assessed the traveling performance of the fabricated wheel on the rigid ground and the sandy soil. The attained net traction and slip ratios approach the values of more complicated, expensive and heavier wheels that were used in farming and planetary exploration. Consequently, the wheel enables the wheeled locomotive to do missions on sandy soil with no trouble.

## Introduction

In human history, making the wheel is one of the most outstanding engineering achievements. Although the exact origin of the wheel is unknown, according to one theory, Sumerians invented the first wheeled chariot more than 5000 years ago^1^.

Since rigid wheels have been the most common, simple, stable and efficient method of locomotion on flat rigid surfaces due to keeping rolling diameter and cross-section at any loading conditions. The static friction of wheels with the rigid ground through rolling is the superior consideration to produce satisfactory traction for vehicles. Consequently, the automotives, mobile robotics and the missions of a rover in planetary explorations are based mainly on the wheeled locomotive.

Because of terrain difficulties such as loose soil that represents the majority of the earth and a crater of the planets, the wheeled locomotive confronts many troubles that cause the traction failure of rigid wheels. The sinkage of a rigid wheel in loose soil destroys static friction and generates higher slip ratios. The static friction dominates when the wheel starts to rotate on the ground. Without static frictional force, a rigid wheel loses grip and cannot move forward at sand sinking case, rotation without any linear displacement.

The sinkage is the vertical distance between a soil’s un-deformed surface and the point located directly beneath the wheel center. Meanwhile, the slip ratio is defined by the ratio of the difference between the wheel circumferential velocity and the wheel moving speed to the wheel circumferential velocity^2^.

To augment traction and overcome terrain difficulties, amending suspension systems, introducing unusual wheels , rigid with grousers or flexible, and using special locomotion systems such as tracks, multiple wheels, legs and screw propelling have been the main developing aspects.

The rigid wheel with the grousers is a possible solution to increase traction force due to grip on loose soil. The Mars rovers Spirit, Opportunity, and Curiosity have all featured grouser wheels in order to satisfy mass and volume constraints imposed by space missions^3^.

Reducing sinkage is the major part of achieving optimal performance. Therefore, flexible wheels increase the length of the contact patch without increasing the wheel size to improve tractive performance^4^. The flexible wheels include pneumatic and metal wheels. Farm tractors used heavy pneumatic tires to fix the soil and attain the mission. Meanwhile, the metal flexible wheel of the ExoMars rover on the loose soil is deformed due to loads to decrease the ground pressure on the loose soil surface and traverse the sand dunes.

The spreading out of a vehicle’s weight on the loose soil improves the traction force of track locomotion systems. But, locomotion systems with six wheels or eight wheels have better performance than tracked systems, especially in rugged terrain^5^. Legged robots represent the best locomotion system on rugged terrain. However, legged robots face a number of challenges; a large number of degrees of freedom, a complexity of walking mechanisms, sophisticated control and the higher possibility of failure^6^. The screw-propelled vehicle is distinguished and based on the mass movement of a medium to cope with snow, ice, mud, and swamp^7^.

Combining two or more previous systems was the focusing point for many researchers to utilize the advantages of each. There were examples of the possible combinations that lead to hybrid locomotion systems: leg-wheels^8^, leg-tracks, wheel-tracks, and leg-wheel-tracks^9^.

Based on this concept, the wheel-leg hybrid robot is presented to scout areas that humans find difficult^8^. There are two main categories; wheel on leg/wheeled-leg and leg-on-wheel/legged-wheel systems. The four-leg-wheel rover is like RoboSimian, SherpaTT and ANYmal^10^. MAMMOTH (Mars Analogue Multi Mode Traverse Hybrid) and ATHLETE (All-Terrain Hex-Limbed Extra-Terrestrial Explorer) with six legs-wheels and more active degree of freedom are multipurpose rovers particularly for planetary exploration^11^. The aforementioned rovers have wheels installed at the end of the legs. On the contrary, the DuAxel rover has a simpler structure and consists of four distinguished design wheels that have the ability to transform from wheel mode to leg (paddle) mode by rotating the rim segments^12^. Ref ^13^. presented a multi-legged robot with multimodal wheel design consisting of an arc leg which is a part of the circle. The wheel state occurs when the left half arc structure and the right half arc structure form a circle. Consequently, this hybridization enables the rovers to move on rough terrain and roll fast on a flat surface. Many robots use transformable wheel-leg designs with one degree of freedom (1-DOF), for climbing steps. To adapt to different step sizes and maintain a transformable wheeled robot with two degrees of freedom (2-DOF), named STEP has been proposed^14^.

The fusion between leg and track locomotion to increase mobility in unstructured environments and soft terrains is an interesting point^15^. Leg track hybrid is fairly popular for difficult environments, provided that speed and energy efficiency are not crucial. There are many ways for combing legs and tracks, like iRbot-SUGV and TitanX^9^. To achieve the previous goals with faster speed and higher energy efficiency, the ComoRAT is driven by four motors and has three options for locomotion, including wheels, tracks or a combination of the two to fulfill a mission^16^. In wheel track hybrid robots, the relative position of tracks and wheels or the track shape can usually be changed to enable or disable wheel contact with the ground, like Helois VI and Galileo wheel^9^.

Snake robots have the advantages of terrain adaptability over wheeled mobile robots and traditional articulated robot arms because of their limbless thin body structure and high flexibility. They have extensive applications in tasks such as rescue, disaster recovery, inspection and minimally invasive surgery^17^.

Snakes can use almost their full body length to apply forces to the ground and produce traction on the deformed and un-deformed grounds. The basic patterns of the snake’s locomotion are lateral undulation, sidewinding, concertina and rectilinear. Snakes change their locomotion patterns in response to the environment^17^. Lateral undulation is one of the most used locomotion strategies in nature, among others including legs, creeping and flying. Lateral undulatory locomotion is widely adopted by snakes for slithering, by microorganisms, fishes and snakes for swimming, and by sandfish lizards for traveling in sand^18^. In this wave-like pattern of movement, all parts of the snake's body move at the same speed by sliding contact or sliding friction with the ground. The parts continuously move from side to side perpendicular to the direction of forwarding motion (Supplementary video 1).

In considering the astonishing lateral undulation locomotion and the ordinary wheel geometry, the study introduces a new design to overcome the limits and the traction failures of rigid wheels on the loose soil without adding lugs or grousers on the wheel's surface^19^.

The new wheel is affordable and replicates lateral undulation movement when it rotates to propagate waves and moves forward like snakes and limbless creatures to enable traversing the deformed and un-deformed ground. In addition, the study presents the full description of the design and the generating serpentine curves of the new rigid wheel and investigates the surface modeling of the recommended corrugated sheet to fabricate the wheel. The experimental work uses a simple wheel tester to assess the traveling performance of the built wheel on different grounds.

### Analysis and design of the suggested wheel

**Figure 1:**
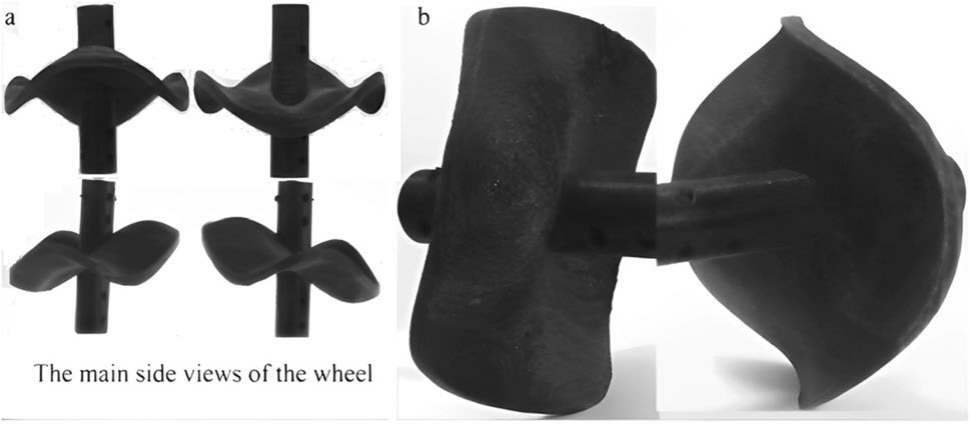
The suggested wheel (**a**) the main views (**b**) the front and rear perspectives on the rigid ground.

This section illustrates the main attributes and the distinguished perimeter of the suggested wheel and studies the effect of a serpentine curve on the velocity analysis. The full description of the wheel and the recommended method to fabricate it will be explained. Figure 1 illustrates the built wheel that will be used in the study. The main views shown in Fig. 1a illustrate serpentine curves of the perimeter. The front and rear perspectives of the wheel shown in Fig. 1b illustrate its point contact area with the rigid ground as conventional types.

Besides, the experimental test layout and the facilities to assess the fabricated wheel are explained. The experiment using simple tests will be conducted to classify the collected soil and define its properties and the size of particles.

#### The Basic mechanics of lateral undulation and the velocity analysis of the new wheel

Figure 1 illustrates the wheel and reveals the distinguished circumference (perimeter). The edges of the wheel follow the serpentine curves of the snake's movement during its rotation. Therefore, the study referred to the analysis of the lateral undulation and oscillation of the snake’s locomotion to describe the mechanics and get the velocity components of the new wheel.

**Figure 2:**
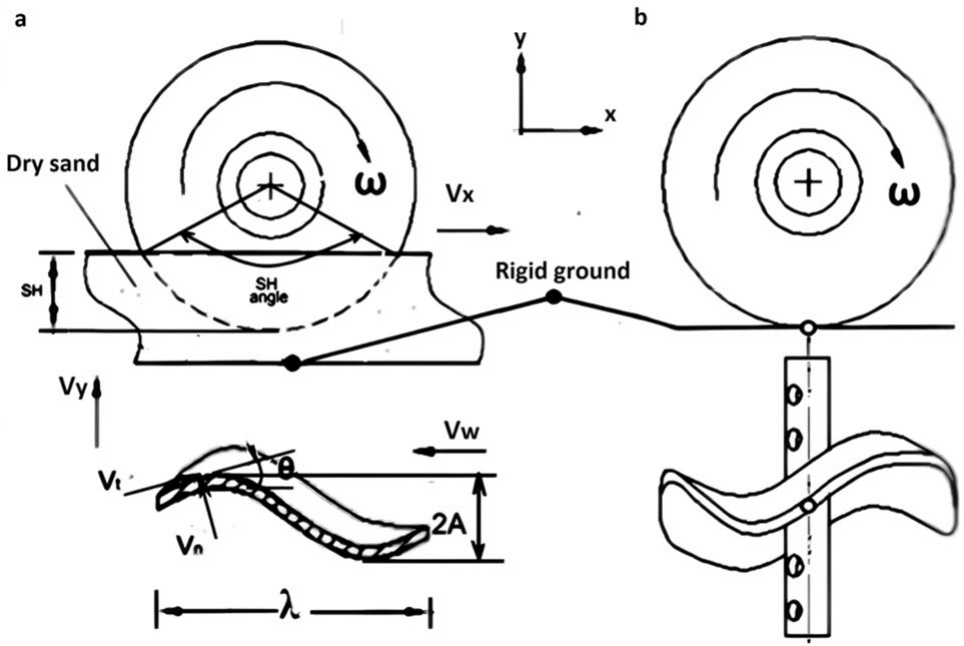
The principle velocities of the wheel on, (**a**) loose soil, and (**b**) rigid soil.

Through sinking in the sand, the wheel behaved as snakes and generated continuous waves that are propagated backward along the wheel or (snake body from head to tail) as shown in Fig. 2a. The body waves produce interaction forces between the wheel (snake) and irregularities in the surface that push the body forward. The body moves from side to side simultaneously to produce forward motion. This oscillation has tangential (${V}_{t}$) and normal (${V}_{n}$) velocity components relative to the forward velocity direction (${V}_{x}$) as shown in Fig. 2a^20,21^. The net result of the lateral oscillation cancels the normal force and the tangential components for both sides are in the same direction, parallel to the direction of forwarding motion.

In the case of sand sinking, ($y$) the displacement from the mid-line of the interaction sinusoidal curve shown in Fig. 2a is defined by the following^21^.

1 $$y = A.\sin \left( {\frac{2.\pi }{\lambda } \left( {x + v_{w} } \right)} \right)t$$

where, A is the amplitude of the sinusoidal curve, λ is the wavelength and x-direction is perpendicular to the axis of the wheel rotation and ${V}_{w}$ is the oscillation velocity of the generating lateral undulation when the wheel rotates with $\upomega$ cycle/s during sinking in the sand.

2 $$V_{w} = \lambda \times {\upomega } \times C_{f}$$

where, ${C}_{f}$ is the correction factor of the frequency that related to the sinkage height of the wheel and the confronting angle (SH) as shown in Fig. 2a.

3 $$C_{f} = 360^{o} /\left( {SH} \right)$$

The velocity of each point of the wheel that interacts the soil is resolved into the transverse (${V}_{y}$) and forward (${V}_{x}$)) components. The transverse or lateral speed the direction is defined by the following equation^21^.

4 $$V_{y} = \frac{\partial y}{{\partial t}} = \frac{{2A\pi v_{w} }}{\lambda } .cos\left( {\frac{2\pi }{\lambda }\left( {x + V_{w} t} \right)} \right)$$

**Figure 3:**
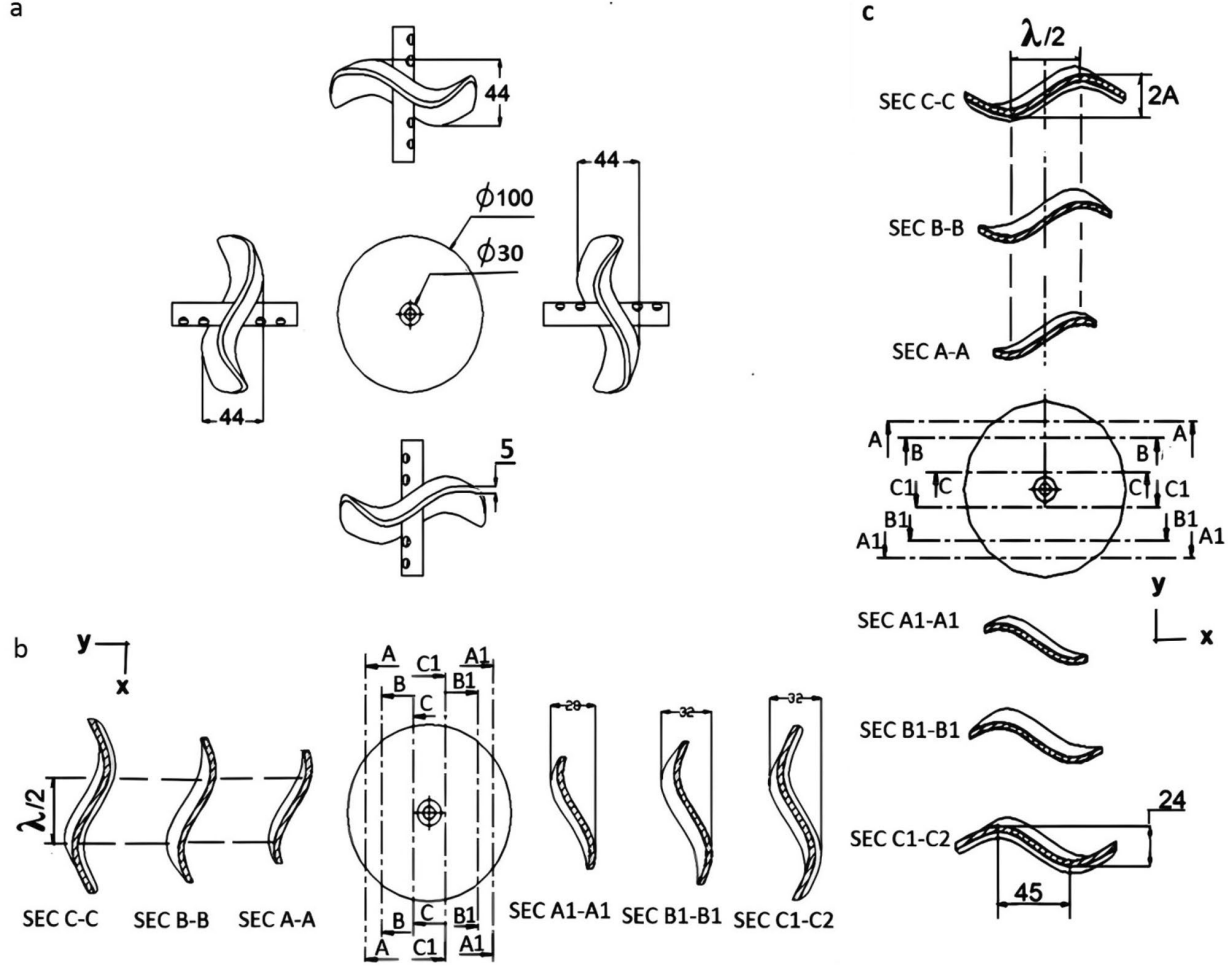
The wheel design, (**a**) the main views and outer dimensions, (**b**) the cutting levels at y-axis, and (**c**) the cutting levels at x-axis the main views and sections of the wheel.

The angle between the forward velocity direction (${V}_{x}$) of the wheel and the instantaneous orientation of the serpentine curve (θ) is shown in Fig. 3-a. and can be obtained by differentiating^21^ Eq. 2.

5 $$\tan \left( \theta \right) = \frac{dy}{{dx}} = \frac{2A\pi }{\lambda }.cos\left( {\frac{2\pi }{\lambda }.\left( {x + V_{w} } \right)} \right)t$$

Thus, the forward velocity is

6 $$V_{x} = \frac{\partial x}{{\partial t}} = \frac{{V_{y} }}{tan\left( \theta \right)}$$

The equations do not consider the effect of the granular frictional fluid during traversing. Sand is usually classified as granular material. It takes the shape of its container as liquids do and one grain of sand is solid.

In the case of moving on the rigid soil, a contact area of the wheel when interacting with the flat ground is similar to rigid wheels, pertaining circularity and the point of contact with the ground as illustrates in Fig. 2b

There is a point of contact between the wheel and the rigid ground and not transverse velocity ($${V}_{y}$$). The forward velocity ($${V}_{x}$$) is the same as the circumferential speed ($${V}_{d})$$ and defined by

7 $$V_{x} = V_{d} = \pi \times D \times \omega$$

where, D is the diameter of the wheel.

#### The wheel design and the serpentine curve

The author presents the main dimensions, detail design and the main attributes of the serpentine curves of the wheel. The diameter, total breadth, thickness and curvy outline of the perimeter of the wheel are shown in Fig. 3a. The curvy outlines or perimeter of this wheel resemble the serpentine curve that can be approximated to a sinusoidal function (Y)^20^.

8 $$Y = A.sin\left( {\frac{2.\pi }{\lambda }.X} \right)$$

So, the wheel attains the lateral undulation gait when it rotates and traverses across the deformable soil.

By using the cutting levels at the elevation view of the wheel (A–A),(B–B) and (C–C), the resultant cross sections at 10, 20 and 40% of the wheel diameter illustrate the sinusoidal curves at the four edges of the wheel as shown in Fig. 3b,c. Consequently, the values of the wavelength (λ) and the amplitude (A) that describe the curves are demonstrated.

#### The corrugated sheet design

**Figure 4:**
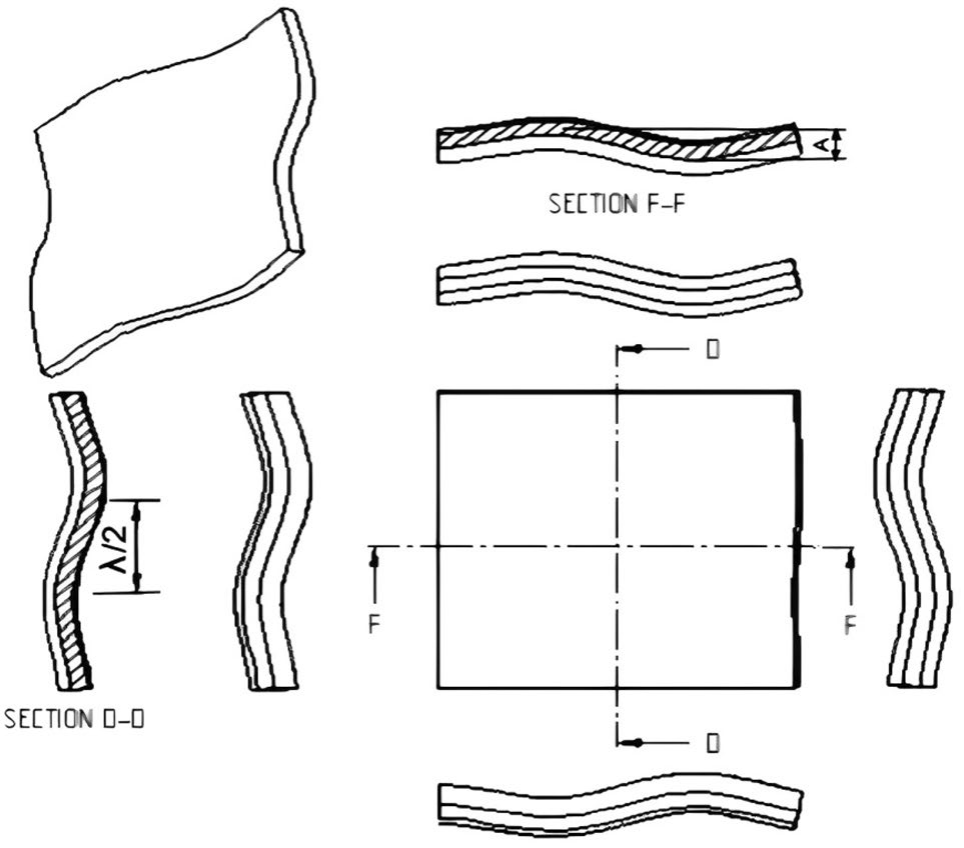
The design of the specific corrugated sheet.

This section introduces the detail design of the recommended corrugated sheet to form the wheel. The corrugation resembles the wavy shape of the lateral undulation bilaterally. This sheet has sinusoidal profiles along its four sides as shown in Fig. 4. The parameters that characterize the serpentine or sinusoidal curve are the wavelength $(\uplambda )$ and the amplitude (A). Based on the observations of various types of snake locomotion, heavy snakes protrude slowly making a larger radius of curvature whereas lean snakes move faster by making a smaller radius of curvature^21^. The amplitude to wavelength ratio of the limbless animals when performing lateral undulation locomotion is within from 0.05 to 0.5.

Due to the empirical and experimental results of the snake and sandfish that move within the granular medium, the maximum forward speed and optimum "S" shape correspond to the ratio A/$\uplambda$=0.2 because of enlarging speed and lowering the resistive force of the undulation locomotion^21^. The corrugated sheet profile satisfies the 0.2 ratio as shown in Fig. 4, sections D–D and F–F. The surface geometry (Sf) of the recommended corrugated sheet is defined by the following equation.

9 $$Sf = A.\sin \left( {\frac{2.\pi }{\lambda }.X} \right) + A.{\text{sin }}\left(\frac{2.\pi }{\lambda }.y\right)$$

Then, the wheel is obtained by cutting out a circular shape of the sheet.

### Experimental work

The experimental work proposes to assess the new wheel's performance. The slip ratio and the net traction are the main parameters for describing performance. Besides, the study assigns the soil conditions and the size of sand particles. To achieve the objective, the study used the single wheel tester to measure the actual forward speed (V_x_) and the net traction and pushing forces of the built wheel on the sandy soil. It is fabricated by a 3D printer and made of plastic (PLA) as shown in Fig. 1.

#### Soil conditions

The author collects the used soil from the top surface of the sandy beach of Port Said. It is located at the north-eastern part of Egypt, which lies at the most northern gate of the Suez Canal^22^. The Soil can be described by the relative abundance of solid primary particles it contains. These particles, depending on their size, can be classified as sand, silt, or clay. Therefore, the study defines the relative proportion of the primary particles or the soil texture of the used soil. It is crucial to assess land use and the built wheel.

The study performs a rapid test based on sedimentation to separate sand, silt and clay and give a general idea of what your soil texture could be. Using the jar soil test is preferred instead of complicated ways. The test procedures are in four steps: filling the jar with half of the soil, pouring the water into 2/3 of the jar and shaking it roughly for more than 10 min. The final step is placing the jar in place for 2 days to resolve the main components of the soil into layers. The soil particles settle out according to size. After one minute, mark the level of sand, after two hours mark the silt depth and after two days mark the clay depth. By measuring the height mark and the thickness of the sand and silt clay layers, the study calculates the percent of the sand, silt and clay and defines the soil type. The soil is classified as the sandy type with a very low amount of clay and silt^23^. By sieve analysis test at the soil mechanics lab at Port Said University, the sand exceeds 99% and the particle diameter ranges from 0.2 to 0.5 mm. The sandy soil is dry and the bulk density is 1.5 g/cm^3^.

#### Experimental setup

**Figure 5:**
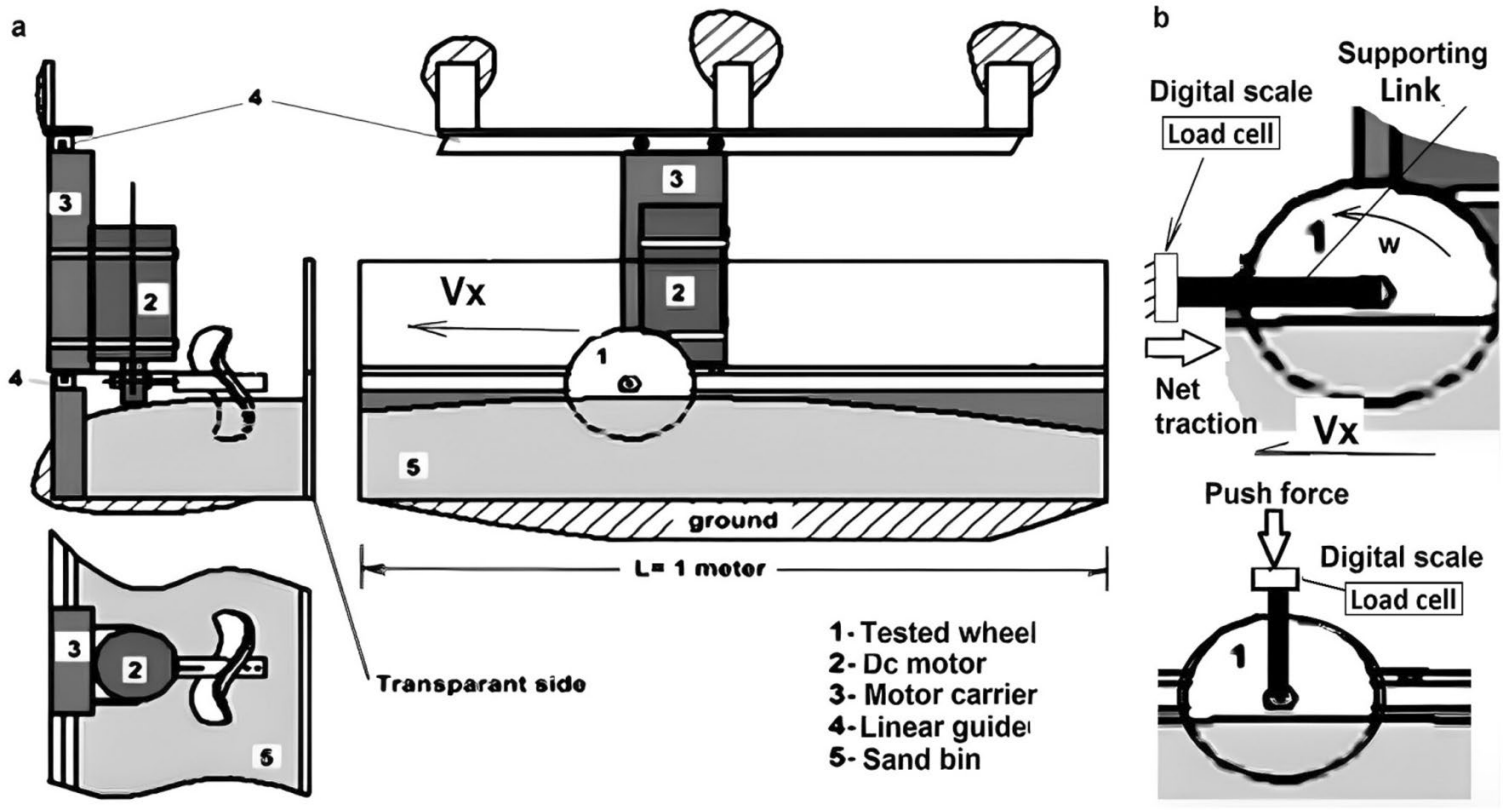
The experimental setup of the wheel test bed, (**a**) forward velocity (**b**) Push and net traction forces.

The layout of the single wheel test rig is shown in Fig. 5a to estimate the forward traveling speed (${V}_{x}$) of the built wheel. The single wheel tester consists of the following items. The built wheel item (1) is assembled with the geared dc motor (12VDC-right angle) item (2) and the carrier item (3). To guide this assembly along the x-direction, there is the linear motion guide item (4). The main dimensions of the used bin item (5) are 1 m long, 0.25 m in width, and 0.12 m in height. It is filled uniformly with loose soil and has a transparent side that is made of an acrylic sheet. After placing the wheel in the sand at the prescribed level, 10, 20 and 40% sinkage height, the study estimates its forward velocities by measuring the time to run 1 m. Figure 5b illustrates the simple traction wheel tester to measure the net traction force. It is based on a single link device to support the rotating wheel in its place in the sand. The study adds the strain gauge load cell (5 kg) straight bar 75 mm long of the digital scale) at the end of the link and performs this test at 10, 20 and 40% sinkage height. The recording data of the digital scale (load cell) is the net traction force (NT). Besides, the required force (Fp) to push the built wheel into the sandy soil is measured. The experimental tests are performed on three trials to estimate the average forward speed, the push and net traction forces. Each trial was conducted under identical soil conditions. The frequency of the trials is the same as^24^.

#### The operating and testing conditions

**Figure 6:**
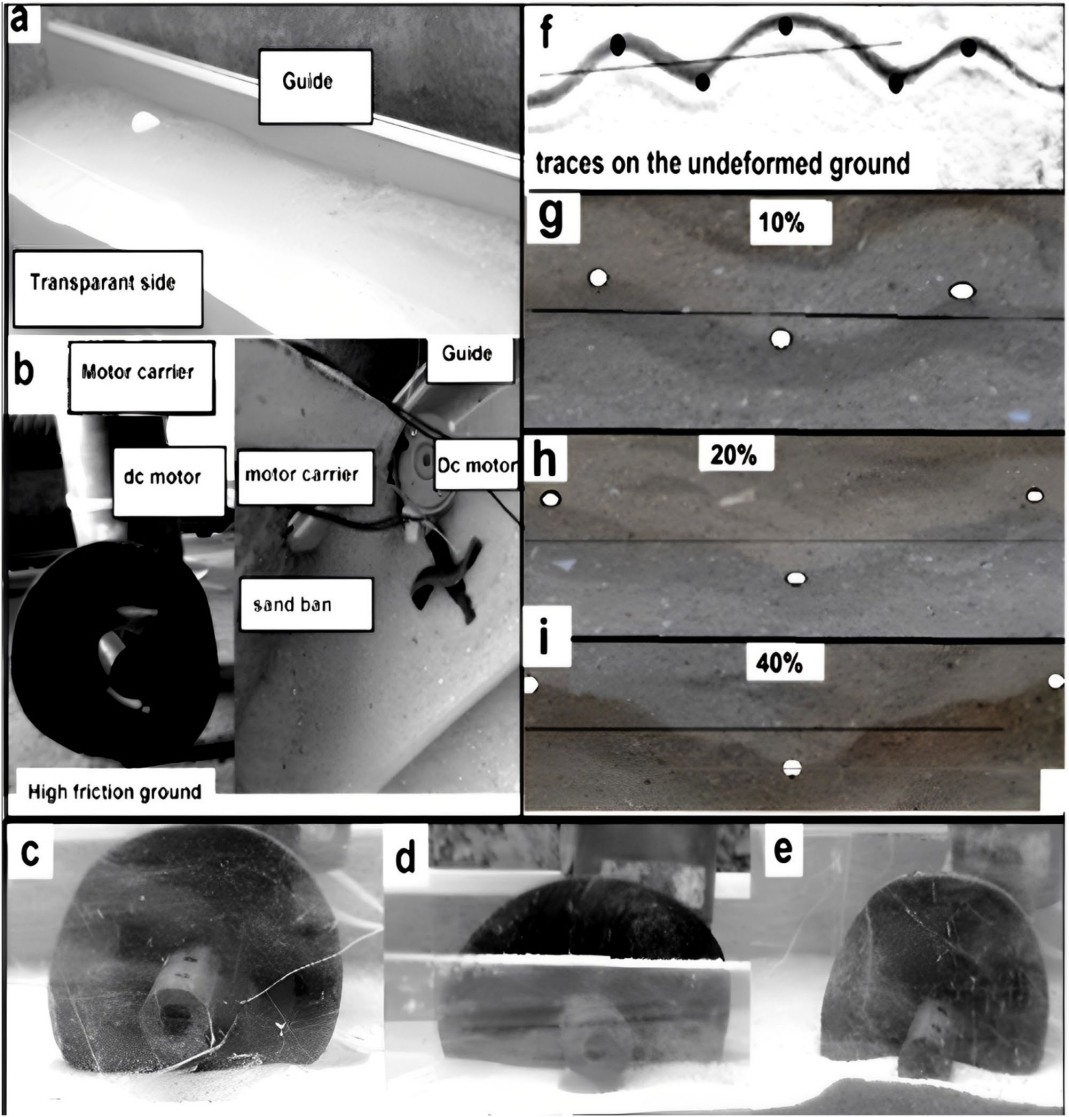
The experimental facilities to assess the built wheel performance (**a**) sand bin (**b**) motor carrier (**c**, **d**, **e**) sinkage heights of the wheel (**f**, **g**, **h**, **i**) The wheel's traces.

The main tools and the experimental facilities to perform the assessment test of the built wheel are shown in Fig. 6. Figure 6(a) illustrates the sand bin and the transparent side. The equipped DC Motor with the tested wheel is shown in Fig. 6 (b). At 10, 20 and 40% sinkage height, Fig. 6c,d,e investigates the built wheel when immersing and rotating in the sandy soil.

The revealing of the wheel's ability to produce lateral undulation is proved by detecting its traces on the ground. During traversing the flat ground, the built wheel imprints the sinusoidal curve traces as shown in Fig. 6f. Figure 6g,h,i shows the traces of the wheel through traversing the sandy soil at 10, 20 and 40% sinkage height. The wheel is never in contact with the bottom of the bin. The solid points in Fig. 6f,g,h,i represent the peaks of the sinusoidal curves of the traces to define amplitude to wavelength ratios.

Table 1 illustrates the main geometrical parameters of the built wheel, the experimental test condition as the rotating speed and the soil conditions and the applied carrying load that the half of the motor weight.

**Table 1:** The experimental test conditions.

The geometrical parameters of the built wheel	Diameter (mm)	Thickness (mm)	Breadth (mm)	Weight (g)
	100	5	44	80
Soil conditions	Soil type	Bulk density (g/cm^3^)	Soil moisture	
	Sandy soil 99% sand	1.5	0%	
The operating conditions	The static load capacity (N)		The rotating speed, $\upomega$ (rpm)	
	8		20	

**Figure 7:**
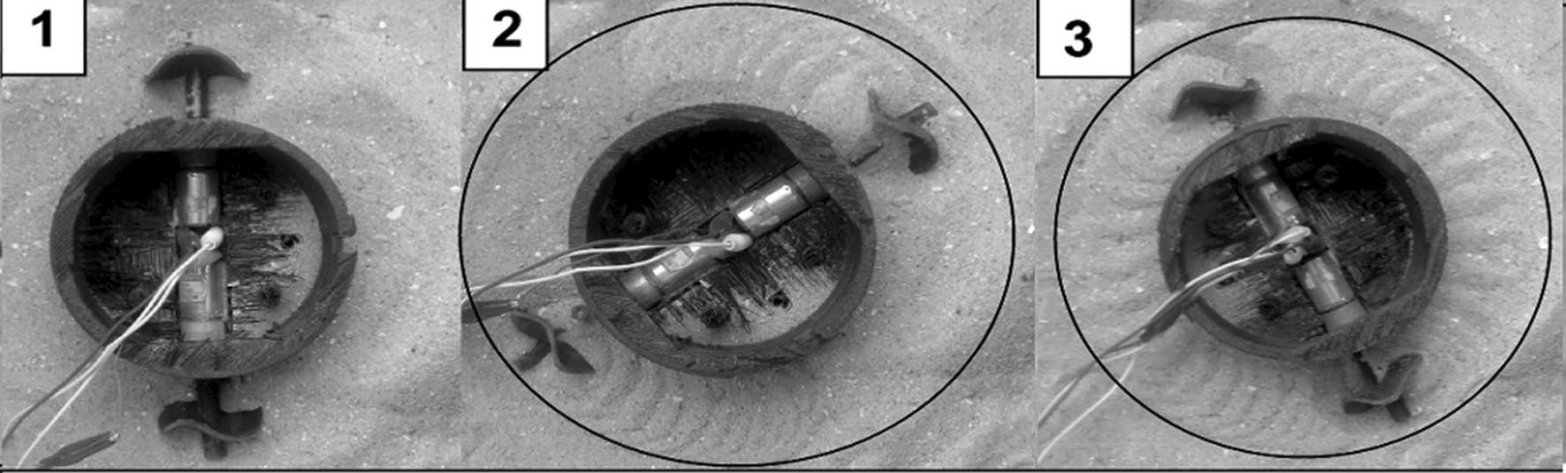
The two fabricated wheels platform through turning on the sand soil and the imprinted traces.

In addition, the built wheel has a turning ability in the sand and does not confront any restrictions. The study builds the two wheeled platform shown in Fig. 7. It consists of two dc motors (JGA25-370 DC gear motor) that connect directly with the 50 mm diameter wheels and operate by using the differential drive to accomplish the full revolution on the sand. The Imprinted traces shown in Fig. 7 resemble rotating snakes.

## Results

Table 2 illustrates the estimated actual traveling velocity of the wheel V_x_ from the experimental tests shown in Fig. 6a at different sinkage heights. Then, to define the performance of the wheel, the wave efficiency $\upxi$ and the slip ratios (S) are calculated by the following equations.

10 $$\xi = \frac{{\left( {V_{x} } \right) experimental }}{\pi \times D \times \omega }$$

11 $$S = 1 - \xi$$

**Table 2:** The assessment values of the introduced wheel.

	Travelling velocity, ${V}_{x}$ (mm/s)	Slip ratio, S	$\mathrm{A}/\uplambda$, Of the traces	Wave efficiency $\upxi$	Push force, Fp (N)	Net traction force, NT (N)
Rigid ground	98	0.02	0.1	0.98	–	–
10% sinkage heigh (sand)	85	0.15	0.11	0.85	8	3
20% sinkage heigh (sand)	75	0.25	0.12	0.75	14	7
40% sinkage height (sand)	40	0.6	0.14	0.40	20	9

The calculated data and the amplitude to the wavelength ratios ($\mathrm{A}/\uplambda )$ of the detected traces shown in Fig. 6f,g,h,i are shown in Table 2. Besides, the net traction (NT) and pushing forces (Fp) of the built wheel are measured by using the single wheel test shown in Fig. 6b.

On the rigid ground, the traveling velocity (V_x_) of the suggested and ordinary wheels with a similar diameter and thickness are 98 mm/s and the calculated sip ratio is 0.2. In the case of the sand sinking, the ordinary wheel did not move forward or traverse and the slip ratio is 100%.

### Wheel cost analysis

The cost of rapid prototyping of the plastic wheel is low and does not exceed 10$ by using a 3D printer, MakerBot Replicator Z18. For mass production fabrication or using metallic materials, the study recommends sheet metal forming that depends on the introduced corrugated sheet shown in Fig. 6. Consequently, the cost is reduced considerably.

## Discussion

The role of biological inspiration differs from common locomotive systems. The design did not replicate the morphology, configuration, or arrangement of the legs of impressive animals. The outline of the wheel is inspired by a serpentine curve that represents the snake’s forward gait, lateral undulation. Therefore, this study formed a simple geometry that had the specific amplitude to wavelength ratio to enable the rigid wheel to traverse sandy soil by self-propelling as a limbless creature. Meanwhile, ref ^25^. introduced a wheel with a curved surface and tooth edge to traverse muddy soil by gripping and increasing the contact area. The bi-cubic spline surfaces that connect the adjacent tooth with the hub of the toothed wheel structure were the method to form its complicated (toroidal) surface^25^.

The diameter and thickness are the geometrical parameters for the ordinary rigid wheel. Meanwhile, the suggested wheel has an extra parameter, amplitude to wavelength ratio, that reflects on the breadth but does not affect the sinkage height as the thickness or diameter. In the case of rigid ground, it keeps circularity and single point contact regardless of the payload changing like ordinary rigid wheels. Furthermore, it does not confront difficulty through the move on the loose soil by accomplishing the lateral undulation through sinking and traversing on the sand. The self-deformation of the soil and interaction with the environment is the operating concept of the suggested wheel, instead of gripping the loose soil using the lugs of the grouser wheel or changing the shape of the flexible wheel to match the contour of the ground.

The wheel gave self-propelling as screw vehicles^26^. However, its axis of rotation is perpendicular to the forward speed, not parallel as the screw-based locomotive that was restricted through moving on dry sand and rigid ground. Dry sand is a known challenge for screw-vehicles because it has minimal cohesion and high frictional properties that cause poor performance and decrease the drawbar pull capacity^26^. The self-propelling ability encourages designers to use the suggested wheel in an unmanned ground vehicle, UGV, instead of adding complex hydraulic and electrical propulsion systems in order to simplify the control system and minimize the energy demands for propelling off-road vehicles^27^.

Overcoming the main drawbacks of snake robotics such as slow speed and sophisticated control to generate sinusoidal curves are advances of the wheel. Its mechanical design is simpler and has a single degree of freedom instead of the multiple links with a higher degree of freedom of snake robotics^16^.

In just two-stages, the wheel design would be built. Firstly, making an unusual corrugated sheet with a purpose and shape differs from conventional types. Finally, cutting out a circular shape of the sheet is the last stage to get the wheel. The study is digging out the best fit amplitude to wavelength ratio from the empirical observation of sandfish to form a sinusoidal profile of the corrugated sheet^21^. The study fabricated the two sizes of the wheel by 3d printer. The 100 mm diameter was assessed by a single wheel tester and the 50 mm diameter was assembled in the two-wheeled system.

During the experimental work, the traces of the built wheel on the rigid and sandy ground are sinusoidal curves that resemble the lateral undulation movement of snakes. With the wheel sinkage height increasing, the amplitude to wavelength ratios of traces varied and became higher, and the calculated slip ratios and the measured forces increased. The rolling friction coefficient between the guide and the rollers is not considered due to the stationary state of the wheel carrier when measuring the forces.

The study focused on the slip ratio and the net traction force, which represent the main assessments to describe the behavior of the wheeled locomotive. According to the outcomes, the wheel's performance on the rigid flat ground approaches the conventional rigid wheel. On the sandy soil where the conventional rigid wheel fails due to the higher slip ratios, the introduced wheel succeeded in rotating and moving forward on the sand at different sinking levels and had the turning ability. The resulting average slip ratios at 10 and 20% sinkage height are within the ideal range (0.1–0.45), rising phase. At 40% sinkage height, the slip ratio does not exceed the maximum limits of the transitional phase (0.6). The slip ratio can be controlled between 0 and 0.8^28^ but when the slip ratio develops to around 1, the wheel will get stuck in loose soil^29^.

To assess the wheel impact, the study carries out the comparison with various sizes and types of rigid, flexible wheels and tires, especially the wheels that are used in planetary exploration, and tabulates their net traction ratios in Table 3. The average values of the net traction ratios are acquired from the curves in the figures of the references approximately. Most of the studies in the table perform three to five experimental trials to get the average values shown in Table 3.

**Table 3:** The net traction ratios comparison.

Wheel name	Wheel type	Diameter/thick/breadth (mm)	Net traction ratios, NT/Fp At (0.15–0.25–0.6) slip ratios, S	Loose soil type
The suggested wheel	Rigid wheel with serpentine curve	100/5/44	0.37–0.5–0.45	Sand
Conventional wheel	Rigid wheel	100/5	Slip ratio (1)-( no movement)	Sand
MER^33^	Rigid wheel with grousers planetary exploration	260/160	(0.2–0.28–0.3)	Mojave Martian
NASA GRC (2)^33^	Rigid wheel planetary exploration	510/180	0.17–0.19–0.21	Loose GRC-1
NASA GRC (1)^33^	Rigid wheel planetary exploration	810/180	(0.25–0.3–0.35)	Loose GRC-3
Lunar rover^29^	Rigid with grousers planetary exploration	180/100	0.15–0.37–0.42	Sand
EXOMARS^4^	Flexible wheel with grousers	300/100	(0.16–0.185–0.2)	Sand
EXOMARS^4^	Flexible wheel with grousers	300/100	0.36–0.45–0.55	MSS-D soil
Novel grouser wheel^3^	Rigid wheel with grousers	220/166	0.25 at 0.2 slip ratio	Sand
Novel grouser wheel^3^	Rigid wheel	220/166	0.13 at 0.2 slip ratio	Sand
165/60R13 tire^31^	Conventional tire agriculture	535/170	0.07–0.1–0.13	Sand
Bridgestone 5–12, 4 ply, lug-M^30^	High-lug agricultural type. tire	550/124	0.37–0.42-NA	Sandy clay loam soil

Nevertheless, the affordability, simpler design and smaller diameter, the built wheel fulfilled the adequate net traction ratios near to the values of the grouser and flexible wheels for planetary exploration and lunar rover^29^. According to the experimental results on agricultural tractor tires that were acquired by ref.^30^, the wheel could be included in farm tractors instead of the heavier and larger lug tires^31^. Increasing diameter enhances the net traction more than width. The larger wheel diameter is more beneficial to decrease sinkage and motion resistance^32^.

As shown in Table 3, many references used sand in experimental works, from tires that operate on the earth to rovers on planets. Rovers have experienced high wheel slip mobility problems as the MER Sprit rover, which was embedded in a sand trap at Troy on Mars with a wheel slip of near 1.0^33^ .The jet propulsion laboratory Mars yard performed tests on dune sand surfaces in the Mojave desert^34^. For the previous reasons, the sand is selected to assess the suggested wheel performance.

## Conclusion and future works

The study presented the new simple, light weight, and affordable design to overcome rigid wheel troubles when traversing sandy soil. The proposed design merged the main attributes of the ordinary wheel and the lateral undulation movement. On the rigid ground, the new wheel behaved, traveled and kept the contacting area as ordinary wheels. Though rotating and moving on the sand, the new wheel printed sinusoidal curve traces and gave self-propelling as the limbless mammals. According to the experimental results, the wheel has the ability to achieve the missions of the wheeled locomotive on the sandy and rigid soil of the earth such as farming or through the planetary exploration missions. Modifying suspension systems to assemble the wheels in vehicles and covering them with serpentine-shaped rubber represent the main imitations and motivations for future works. Besides, studying its applicability and performance when using by wheeled locomotive systems and mobile robotics and changing size, material and operating conditions will be interesting points. The study encouraged researchers to utilize this wheel by presenting a full description of its design and fabrication.

## Supplementary Information

Supplementary Video 1.

## Data Availability

All data generated or analysed during this study are included in this published article.

## References

[1] Kenji N, Mizukami N, Kubota T (2012). Prediction of tractive limitations of a rigid wheel on loose soil. J. Asian Electric Veh..

[2] Zhu S, Wang L, Zhu Z, Mao E, Chen Y, Liu Y, Du X (2022). Measuring method of slip ratio for tractor driving wheels based on machine vision. Agriculture.

[3] Nassiraei A, Skonieczny K (2020). Grousers improve drawbar pull by reducing resistance and generating thrust at the front of a wheel. J. Terrramech..

[4] Favaedi Y (2011). Prediction of tractive response for flexible wheels with application to planetary rovers. J. Terrramech..

[5] Hardarson, F. (1998) Locomotion for difficult terrain. *KTH*.‏

[6] Reina G, Foglia M (2013). On the mobility of all-terrain rovers. Indust. Robot Int. J..

[7] Hiram, J., Ramadoss, V., Zoppi, M., & Molfino, R. (2017) Conceptual design of tetrad-screw propelled omnidirectional all-terrain mobile robot. *In 2017 2nd International Conf. on Control and Robotics Engineering (ICCRE)*, pp. 13–17.

[8] Zheng C, Liu J, Grift TE, Zhang Z, Sheng T, Zhou J, Yin M (2015). Design and analysis of a wheel-legged hybrid locomotion mechanism. Adv. Mech. Eng..

[9] Bruzzone L, Giuseppe Q (2012). Locomotion systems for ground mobile robots in unstructured environments. Mech. Sci..

[10] Biswal P, Mohanty PK (2021). Development of quadruped walking robots: A review. Ain Shams Eng. J..

[11] Reid, W., Göktogan, A. H., & Sukkarieh, S. (2014) Moving mammoth: Stable motion for a reconfigurable wheel-on-leg rover. *In Proc. of Australasian Conference on Robotics and Automation*. pp. 1–10‏.

[12] Nesnas IA, Matthews JB, Abad-Manterola P, Burdick JW, Edlund JA, Morrison JC, Anderson RC (2012). Axel and DuAxel rovers for the sustainable exploration of extreme terrains. J. Field Robot..

[13] Ning, M., Xue, B., Ma, Z., Zhu, C., Liu, Z., Zhang, C. & Zhang, Q. (2017) Design, analysis, and experiment for rescue robot with wheel-legged structure. *Math. Prob. Eng*. **2017**, 1–16

[14] Won J (2022). Design optimization of a linkage-based 2-DOF wheel mechanism for stable step climbing. Sci. Rep..

[15] Bruzzone L, Shahab EN, Fanghella P (2022). Tracked locomotion systems for ground mobile robots: A review. Machines.

[16] Gustav, S., Feng L., & Möller B. (2021) A Reconfigurable test platform for developing autonomous articulated pendulum-arm suspension forest machines. *20th International and 9th Americas Conf. of the ISTVS*.‏

[17] Jindong L, Tong Y, Liu J (2021). Review of snake robots in constrained environments. Robot. Auto. Syst..

[18] Zhu L, Yang P, Li F, Wang K, Shui L, Chen X (2021). On the snake-like lateral un-dulatory locomotion in terrestrial, aquatic and sand environments. J. Mech. Phys. Sol..

[19] Nagaoka K, Sawada K, Yoshida K (2020). Shape effects of wheel grousers on traction performance on sandy terrain. J. Terrramech..

[20] Netta, C., & Jordan, B. (2009) Undulatory locomotion. *arXiv preprint* pp. 1–16,.

[21] Yang D, Sharpe S, Masse A, Goldman D (2012). Mechanics of undulatory swimming in a frictional fluid. PLoS Comput. Biol..

[22] Rana Y, El-Rayes A, Sultan Y, Aziz A (2017). Mapping of soil geochemistry in Port Said governorate, Egypt utilizing GIS and remote sensing techniques. Imp. J. Interdiscip. Res..

[23] Whiting, D., Card, A., Wilson, C., & Reefer, J. Estimating soil texture. *Colorado State University Extension Publication,* **214**. (2014).

[24] Yusa J, Sutoh M, Nagatani K, Yoshida K (2012). Traveling performance evaluation for planetary rovers on loose soil. J. Field Robot..

[25] Chen Z, Jinliang G, Xingfa Y (2020). A novel rigid wheel for agricultural machinery applicable to paddy field with muddy soil. J. Terrramech..

[26] Jon FT (2010). A study of omnidirectional quad-screw-drive configurations for all-terrain locomotion.

[27] Czapla T, Marcin F, Roman N (2022). Experimental identification of wheel-surface model parameters: various terrain conditions. Sci. Rep..

[28] Liang D, Deng Z, Gao H, Nagatani K, Yoshida K (2011). Planetary rovers’ wheel–soil interaction mechanics: new challenges and applications for wheeled mobile robots. Intel. Serv. Robot..

[29] Yoshida, K., Watanabe, T., Mizuno, N., & Ishigami, G. (2003) Slip, traction control, and navigation of a lunar rover. *In Proc. of the 7th international symposium on Artificial intelligence*, robotics and automation in Space, Nara, Japan.

[30] Yahya A, Zohadie M, Ahmad D, Kheiralla AF (2006). Net traction ratio prediction for high-lug agricultural tyre. J. Terrramech..

[31] Shinone H, Nakashima H, Takatsu Y, Kasetani T, Matsukawa H, Shimizu H, Ohdoi K (2010). Experimental analysis of tread pattern effects on tire tractive performance on sand using an indoor traction measurement system with forced-slip mechanism. Eng Agric Environ Food.

[32] Liu J, Tang S, Cheng P, Liu S (2012). Influence analysis and evaluation of wheel parameters on motion performance of lunar rover. Inf. Technol. J..

[33] Johnson JB, Duvoy PX, Kulchitsky AV, Creager C, Moore J (2017). Analysis of Mars exploration rover wheel mobility processes and the limitations of classical terramechanics models using discrete element method simulations. J. Terrramech..

[34] Zhou F, Arvidson RE, Bennett K, Trease B, Lindemann R, Bellutta P, Senatore C (2014). Simulations of mars rover traverses. J. Field Robot..

